# A Comparative Evaluation of Student Perceptions of Binocular Loupes and Torchlights in Ophthalmology Skills Training for Undergraduate Medical Students: A Crossover Trial Employing a Mixed-Methods Approach

**DOI:** 10.7759/cureus.98663

**Published:** 2025-12-07

**Authors:** Aparna Singhal, Harish S Agarwal, Arvind Yadav, Sagarika Aggarwal

**Affiliations:** 1 Ophthalmology, Maharaja Agrasen Medical College, Agroha, Hisar, IND; 2 Forensic Medicine, Maharaja Agrasen Medical College, Agroha, Hisar, IND; 3 Pharmacology, Geetanjali Medical College and Hospital, Udaipur, IND

**Keywords:** binocular loupe, clinical skills, competency-based medical education, india, ophthalmology, torchlight examination, undergraduates

## Abstract

Introduction

The Competency-Based Medical Education (CBME) framework in India prioritizes clinical skill acquisition in undergraduate training. Torchlight examination is traditionally used in ophthalmology education, while binocular loupes, offering magnification and stereopsis, may enhance learning. Their effectiveness at the undergraduate level has yet to be systematically compared.

Methods

Third-year MBBS students underwent interactive teaching sessions and were randomly assigned to two groups. Group A performed anterior chamber examination with a torchlight; Group B used a binocular loupe. Groups then exchanged tools. Students and facilitators completed structured Likert-scale questionnaires and provided open-ended feedback, analyzed thematically.

Results

Both modalities were positively received. Torchlight training led to 90% of students affirming improved confidence and skill transfer, most rating the sessions as good or excellent. Binocular loupe training was rated similarly, with 90% reporting confidence and skill enhancement, and 95.7% rating session quality as good. Comparative analysis revealed binocular loupes were considered more interesting (75.4%) and easier for learning (62.3%), with 37.7% finding them handier, though responses on convenience varied.

Conclusion

Torchlight and binocular loupe training each contributed to clinical skill advancement; however, students favoured binocular loupes for interest and ease of learning. Their inclusion, alongside torchlight, could increase engagement and strengthen ophthalmology training in undergraduate curricula.

## Introduction

The implementation of the Competency-Based Medical Education (CBME) framework in India since 2019 has heralded a paradigm shift in undergraduate medical education, focusing not only on knowledge acquisition but also on the development of clinical competence and essential skills [1]. The overarching aim is to ensure that the Indian Medical Graduate (IMG) is well-equipped to serve as the first point of contact in the community, capable of diagnosing and managing common health problems at the primary care level and facilitating early referrals when necessary [2].

Ophthalmology, a core discipline in medical education, is relevant in India because of the high burden of avoidable blindness and visual impairment. According to a nationally representative Rapid Assessment of Avoidable Blindness (RAAB) survey conducted between 2015 and 2019, 92.9% of blindness and 97.4% of visual impairment in India are attributable to avoidable causes [3]. Therefore, early and accurate ocular examination at the primary healthcare level is critical for the timely diagnosis and management of these conditions, directly contributing to the reduction of visual morbidity in the population [4].

Examination of the anterior chamber is essential for detecting sight-threatening conditions such as hyphema, hypopyon, and corneal opacities. However, traditional ophthalmic teaching methods at the undergraduate level often rely on the use of a simple torchlight, which, due to limited illumination and lack of magnification, may fail to reveal subtle or early clinical signs [5]. In contrast, assessment with magnification and binocularity, such as that provided by slit lamps or binocular loupes, offers a stereoscopic and detailed view of ocular structures, facilitating a more accurate diagnosis. While slit lamps have become the standard for postgraduate training and ophthalmology practice, their learning curve and lack of portability limit their widespread use in undergraduate and primary care settings [6].

Binocular loupes, employing straightforward magnifying optics combined with binocularity, present a promising and easily adoptable alternative for undergraduate training. In addition to providing a clear, magnified, and stereoscopic view of the anterior segment, they allow hands-free operations, enabling students to perform clinical examinations and minor procedures more efficiently. Despite their potential, binocular loupes remain underutilized in current undergraduate curricula, where torchlight examination prevails [7].

Recognizing these gaps, the present study was conceived as a medical education research project. This study aimed to compare undergraduate medical students’ learning experience and perceived effectiveness of binocular loupes versus traditional torchlight during anterior segment examination training. The study specifically sought to evaluate both student and faculty satisfaction, feasibility, and challenges that occurred with this teaching tool.

## Materials and methods

This study was conducted in the Department of Ophthalmology after obtaining approval from the Institutional Ethical Committee for Human Research, Maharaja Agrasen Medical College, Agroha, Hisar vide letter no. MAMC/IEC/2025/85. In this crossover experimental study, a mixed-methods approach was employed. In the quantitative strands of the present study, two groups (torchlight and loupe) were randomly assigned for eye examination, and later these experimental groups were crossed over. Structured Likert-scale questionnaires assessing perceptions were given to study participants. In qualitative strands, open-ended feedback was collected and analyzed thematically from participants and facilitators. Integration occurred as both types of data informed the overall evaluation of each teaching method, providing both measurable outcomes and contextual insights for a holistic understanding. Phase III MBBS students came to the department, and their clinical posts were the participants of this study. Students were made aware of the purpose of the study, and those who consented verbally were included in the study. Facilitators, including interns and junior residents who were posted in the department, were also included in the study after their verbal consent to demonstrate and assess their skills.

Preparation of the feedback questionnaire

Feedback consisting of six statements was prepared. A three-point Likert scale, with responses ranging from "Agree (scored as 3)" to "Disagree (scored as 1)," was employed to assess participants’ opinions. The questionnaire was intended to assess the perception of varied aspects of skill teaching sessions, such as attitude, effect on knowledge, quality, and adequacy of the session (Appendix A). Another section of the questionnaire, consisting of three statements comparing both modalities and an open-ended questionnaire for suggestions, was also created. The questionnaire was validated by subject experts and active members of medical education (n = 6) (Appendix B). Reliability was assessed by Cronbach’s alpha coefficient test, which revealed good internal consistency (Cronbach’s alpha = 0.79).

Sensitization of students and faculty

Both students and facilitators were sensitized through an interactive lecture using a PowerPoint presentation (Microsoft Corp., Redmond, WA, USA) and interactive digital tools such as Poll Everywhere (Poll Everywhere Inc., San Francisco, CA, USA) before the commencement of the study.

This was a feasibility-based sample including all available and consenting students in the posting; no formal a priori power calculation was performed. To ensure allocation concealment, sequentially numbered opaque sealed envelopes (SNOSEs) were prepared in advance, with each envelope containing the assigned intervention as per the randomization sequence. These envelopes were securely stored and opened sequentially by the principal investigator at the time of participant enrolment to determine group allocation. Students who performed the initial examination with torchlight were assigned to Group A, and those who performed the initial examination with a binocular loupe were assigned to Group B. A small group of students, with no more than 10 students, was enrolled in one skill training session. One facilitator was allotted to each group of 10 students and taught how to diagnose common clinical conditions affecting the anterior chamber with their features. The topic was the same for both groups. A total of 45 minutes was allotted to each teaching session. The facilitators taught the topic with the help of audio-visual aids, which were prepared under the supervision of the principal author to maintain the uniformity of teaching content. Demonstration was done by respective examination modality, i.e., torchlight and binocular loupe. The skill of examining the ocular surface and anterior chamber was taught by the facilitators by demonstration with the help of the binocular loupe (3.5× magnification), and torchlight (simple, used in normal clinical settings). The students assumed the roles of professionals and clinicians while examining the patients.

Two perceptions (one for the binocular loupe and one for the torchlight) of each student were considered separately. Students who completed both examination modalities were allowed access to a section of the questionnaire pertaining to the comparison of both examinations. Students’ perceptions were taken on a Google form after the completion of each session. The facilitators of the session were also asked to fill in the Google Forms (Google LLC, Mountain View, CA, USA) for their perception.

**Figure 1:**
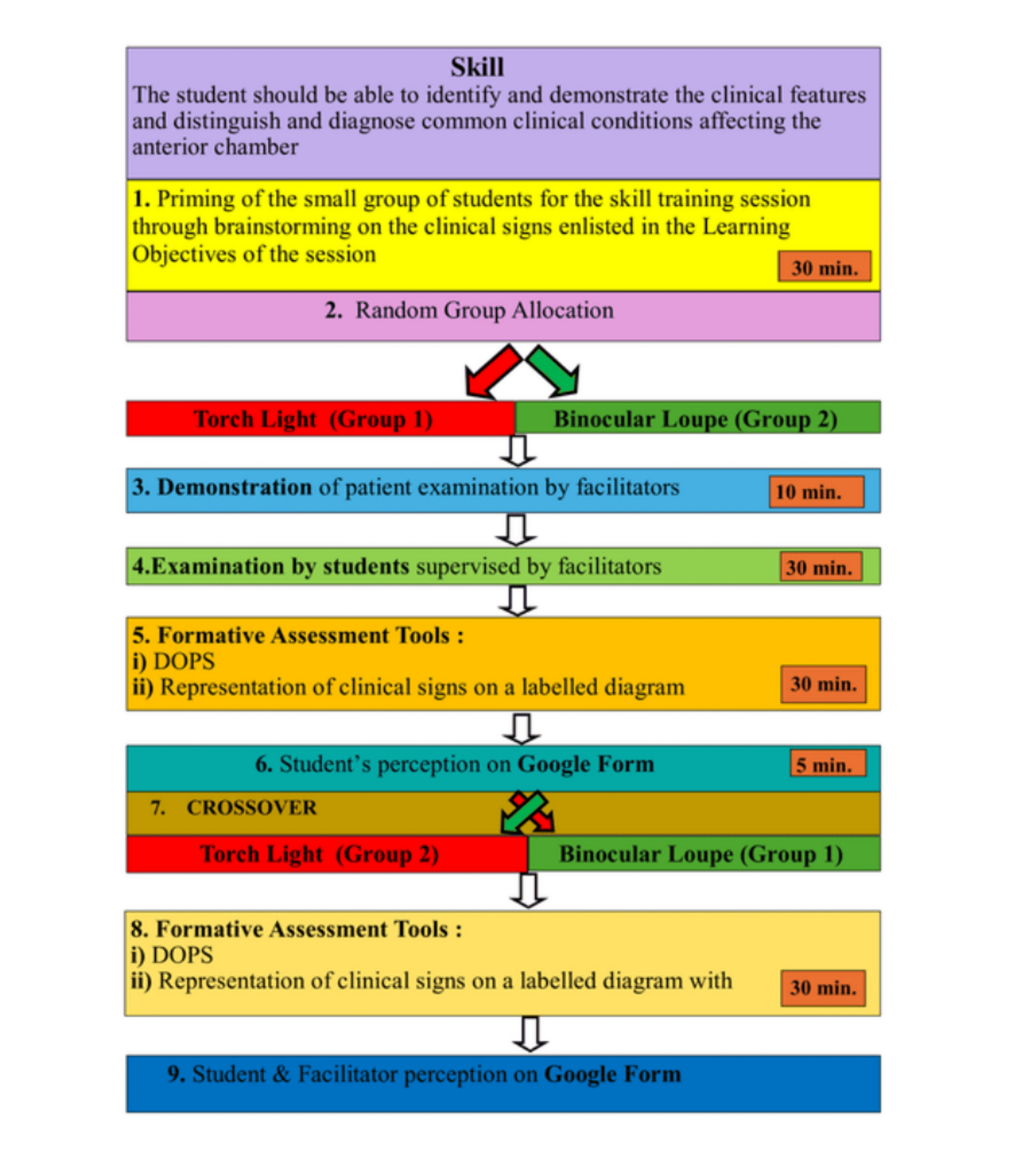
Flowchart illustrating the steps of the skill-teaching session. DOPS: direct observation of procedural skills

A systemic, structured approach was adopted for inductive thematic analysis of qualitative data. An author selected the quote by reading an individual statement. On reading the statement, recurring patterns and terms were identified as keywords. Short phrases or words having these keywords were coded separately into sub-themes. These codes/subthemes were organized into meaningful groups, i.e., themes. An outline of the skill teaching session is given in Figure 1.

## Results

A total of 69 third-year MBBS students and 13 facilitators participated in sensitization training. Patients from the OPD and ward who consented to repeated examinations were included in the session. The study included all the cohorts available to the study.

**Figure 2:**
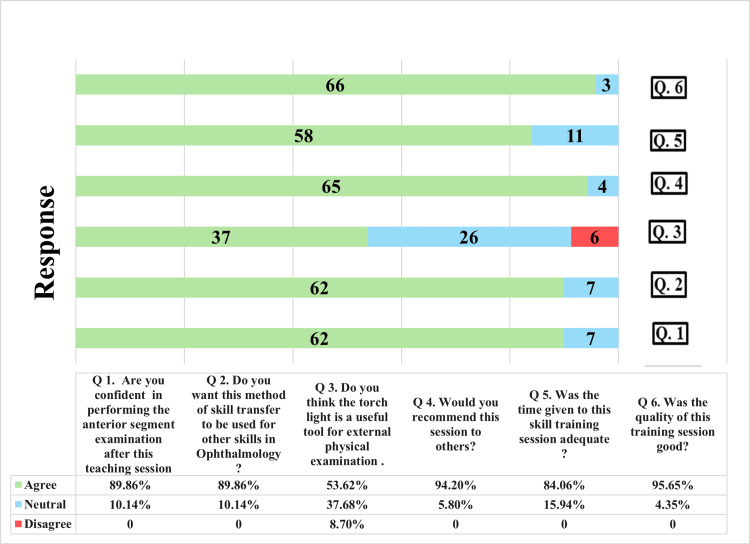
Students’ perception of the torchlight skill training session.

Torchlight sessions were rated highly, with 90% of the students agreeing that they had gained confidence and could transfer skills to another ophthalmic domain. Nearly all participants would recommend torchlight sessions (95.6%) and rate the overall quality as good (95.7%). Only 53.6% (37 out of 69) of the students thought that torchlight was a useful tool for external physical examinations. (Figure 2).

**Figure 3:**
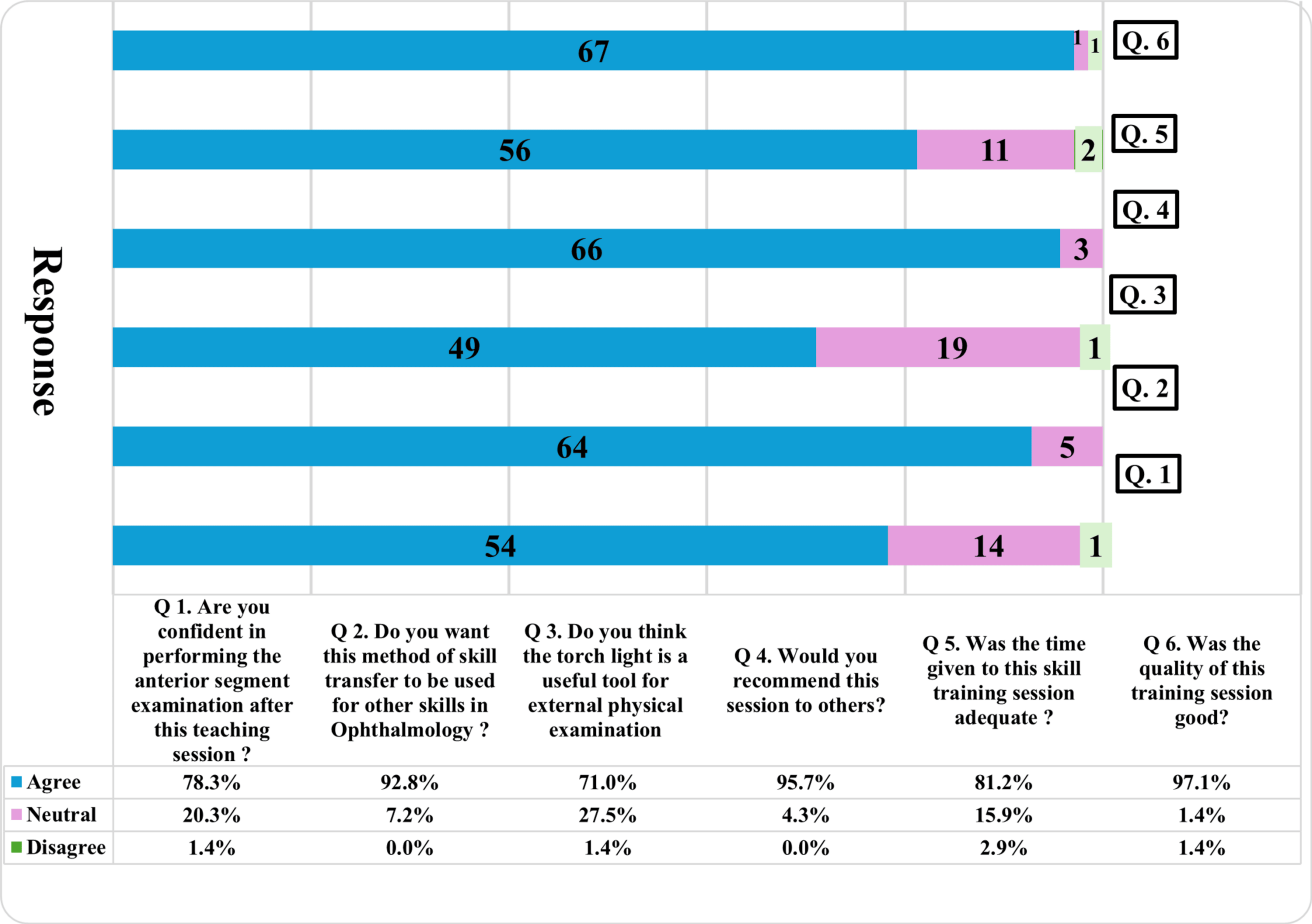
Students' perception of the use of binocular loupes.

Binocular loupe training garnered similarly strong positive responses. Ninety percent agreed on confidence gain and skill transfer within ophthalmology, and 95.7% rated session quality as good. A total of 78.3% (54 out of 69) students performing anterior segment examination with the binocular loupe felt confident in performing the examination after the skill training session. A total of 92.8% (64 of the 69) students wanted this method to be used for learning other ophthalmology skills. Only 71% (49 out of 69) of students thought that the binocular loupe is a useful tool for external physical examination. Although 95.7% (66 out of 69) of students found the quality of the skill training session to be good, only 81.2% (56 out of 69) felt that the time given to the skill training session was adequate. A total of 97.1% (67 out of 69) of the students recommended the skill training session to other students (Figure 3).

**Figure 4:**
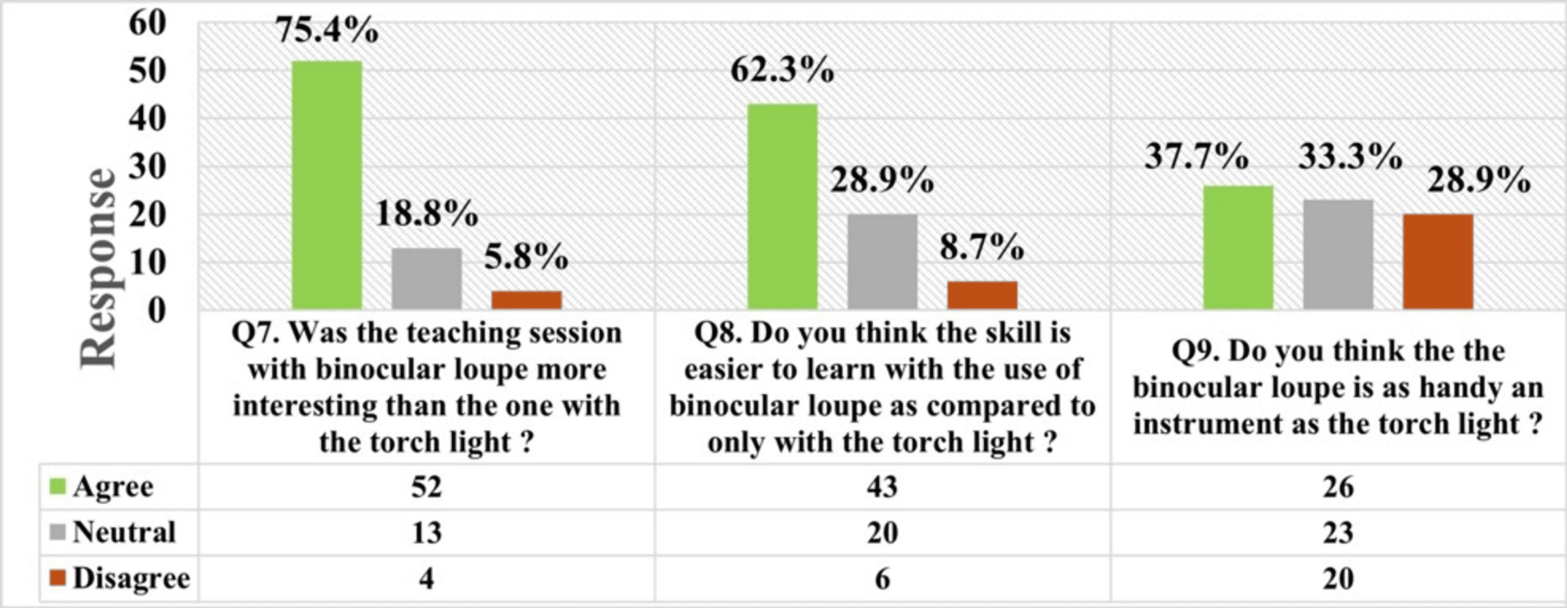
Students’ perception of the skill training at the end of both sessions, comparing the use of the binocular loupe with that of torchlight.

A total of 75.4% (52 out of 69) of these students found using the binocular loupe more interesting as compared to using the torchlight. Around 62.3% (43 out of 69) of students found it easier to learn the skill with the binocular loupe than with the torchlight. Nearly 37.7% (26 out of 69) found the binocular loupe to be as handy as the torchlight, while 29% (20 out of 69) students disagreed with the same (Figure 4).

Only 40 students responded to the open-ended questions. Many of the students were of the view that the binocular loupe scored over the torchlight to examine the anterior segment because of image magnification, increased clarity, easy visibility of the anterior chamber and iris pattern, and better appreciation of the contour of the corneal surface, ulcer margins, and depth of the lesion. However, the proponents of the examination with the torchlight found it easier and more convenient to use. They believed that the identification of most clinical signs with torchlight was comparable to that with the binocular loupe. Other reasons cited for not finding the binocular loupe examination were the difficulty in adjusting the binocular loupe and discomfort for a myopic examiner (Table 1).

**Table 1:** Students’ responses to open-ended questions on the advantages of the examination method.

Theme	Sub-theme	Comment	Response % (n = 40)
Binocular Loupe	Magnification and Clarity	“Magnification is more than torch light”	82
		“Details are observed more clearly”	
	Visualization of Ocular Structures	“Pattern of iris was easily visible”	25
		“Margins of ulcer can be seen easily”	
	Ease and Quality of Examination	“Better appreciation of findings”	10
Torchlight	Comfort and Convenience	“Torch light is easier”	12.5
		“It is not comfortable to visualise the eyes for the examiner who is myope	
		"Examination using torch light need no adjustment"	
	Both are the same	“Clinical signs identified same in both methods”	2.5

All facilitators agreed that the use of a binocular loupe made the session interesting and that the teaching session was executed effectively. They were also satisfied with the use of the binocular loupe as a skill teaching aid. Most believed that the availability of a smaller number of facilitators and inadequate time were the challenges faced during the execution of skill training sessions. They suggested more frequent brainstorming on innovative teaching methods to enhance their ability to teach psychomotor skills (Table 2).

**Table 2:** Facilitators’ responses to open-ended questions on challenges and suggestions for the binocular loupe

Theme	Sub-theme	Comment	Responses % (n = 13)
Challenges	Resource Constraints	“Short of faculty and time”	15.3
	Technical Challenges	“Focusing with the loupe”	46.3
Suggestions	Equipment Needs	“Higher magnification loups”	7.7
	Innovative Teaching	“Training in innovative teaching methods”	38.5
	Positive experience	“No challenge.” “Session more productive”	30.8

## Discussion

This study sought to quantitatively and qualitatively explore undergraduate medical students' perceptions of the use of torchlight and binocular loupes for anterior chamber skill training within an Indian ophthalmology curriculum. The students felt more confident when using torchlights. This high level of confidence may be attributed to its simplicity and widespread availability. Torchlights are easily accessible and do not require additional adjustments, making them convenient tools for initial skill training. A total of 78.3% of students performing the anterior segment examination with the binocular loupe felt confident in performing the examination after the skill training session. This can be justified by the students' recognition of the enhanced visual details provided by magnification. The ability to observe subtle features of the anterior segment, such as the contour of the corneal surface and ulcer margins, might have contributed to increased confidence using this aid. A study conducted on pre-clinical dental students found that loupe magnification significantly enhanced the performance of students, and even students perceived the use of loupes as effective in their training [8].

Our students, too, did not find the binocular loupe very handy, as they were not used to examining the eye with it. A study conducted by medical students advocated that familiarity with the equipment is important for learning psychomotor skills [9]. The belief that this method is useful for external physical examination in patients with dermatological lesions suggests its versatility.

Students inclined towards binocular loupes are likely to prioritize a more detailed and professional approach to anterior segment examination, reflecting a broader perspective on skill development in ophthalmology. As facilitators adopt regular use of binocular loupes in training sessions and the curriculum integrates this tool, all graduates will become competent in the skill, ultimately contributing to a reduction in avoidable blindness in India.

Since the optics of the binocular loupe enhance the quality of ocular examination, many of the facilitators have been using binocular magnification for diagnostic and therapeutic purposes; most of the facilitators were satisfied with the use of the binocular loupe as a skill teaching aid. The main challenges identified in this study were inadequate time and the availability of a smaller number of facilitators. To overcome this time constraint, skill training sessions can be held in evening ward clinics for undergraduate students. To increase the number of facilitators, junior doctors can be enrolled under the supervision of prior sensitization. The facilitators who participated in the study suggested more frequent brainstorming on innovative teaching methods to enhance their ability to teach psychomotor skills.

These findings align with broader literature on medical education, which underscores the importance of learner-centered approaches and the integration of technology-enhanced tools for skill acquisition [10]. Studies have shown that student engagement, satisfaction, and perception of authenticity are strongly correlated with deeper learning and better skill retention [11]. Binocular loupes, by offering a more immersive and detailed view, may enhance the “show-how” and “do” competencies that are central to CBME’s pyramidal model of outcomes. The results of this study also suggest that while torchlights continue to play an important role in primary care and resource-limited settings, augmenting their use with skill sessions involving binocular loupes could offer a pragmatic pathway for elevating the standard of undergraduate education, especially where slit lamps may not be available. As the students carried out both modalities, the effect of one on the other at the time of giving the response could not be ruled out, which is one of the limiting factors of the present study.

## Conclusions

This study highlights how pivotal hands-on experience is in medical education. By introducing binocular loupe training into routine undergraduate ophthalmology teaching, students found themselves more comfortable managing clinical examinations using a binocular loupe. The clarity and magnification offered by the loupe seemed to give learners a new sense of confidence, helping them notice fine details that a torchlight might miss. These insights don’t just improve examination skills - they help future doctors build stronger habits of careful observation and nuanced clinical reasoning, both of which are crucial for patient care. Facilitators echoed these sentiments, noting that sessions felt livelier and students seemed more engaged when using the binocular loupe. Even the challenges, like tricky adjustments or limited resources, were treated as opportunities to refine the teaching process. The positive reception suggests that medical educators should consider making binocular loupe skills a regular fixture alongside torchlight exams, crafting a more well-rounded approach to ophthalmology training. Both methods have their place: torchlights deliver basic accessibility and practicality, while loupes open the door to more advanced skill-building.

The implications reach beyond individual skill labs. By weaving together innovation and tradition, educators can create learning environments that foster curiosity, confidence, and a deeper understanding of clinical work. This not only benefits students while in school, but it also prepares them for real-world challenges, ensuring that their foundation in ophthalmology is solid and adaptable. Going forward, future studies could explore how such tools affect long-term learning and patient outcomes, keeping the focus on ongoing improvement and adaptability. Above all, this research underscores the importance of expanding teaching techniques to meet the ever-changing needs of medical education. By embracing feedback, technology, and collaborative problem-solving, faculty and students can work together to build the kind of clinical competence that truly makes a difference. It’s a journey-one where each new tool, each lesson learned, strengthens the bridge between theory and practice.

## Figures and Tables

**Figure 5:**
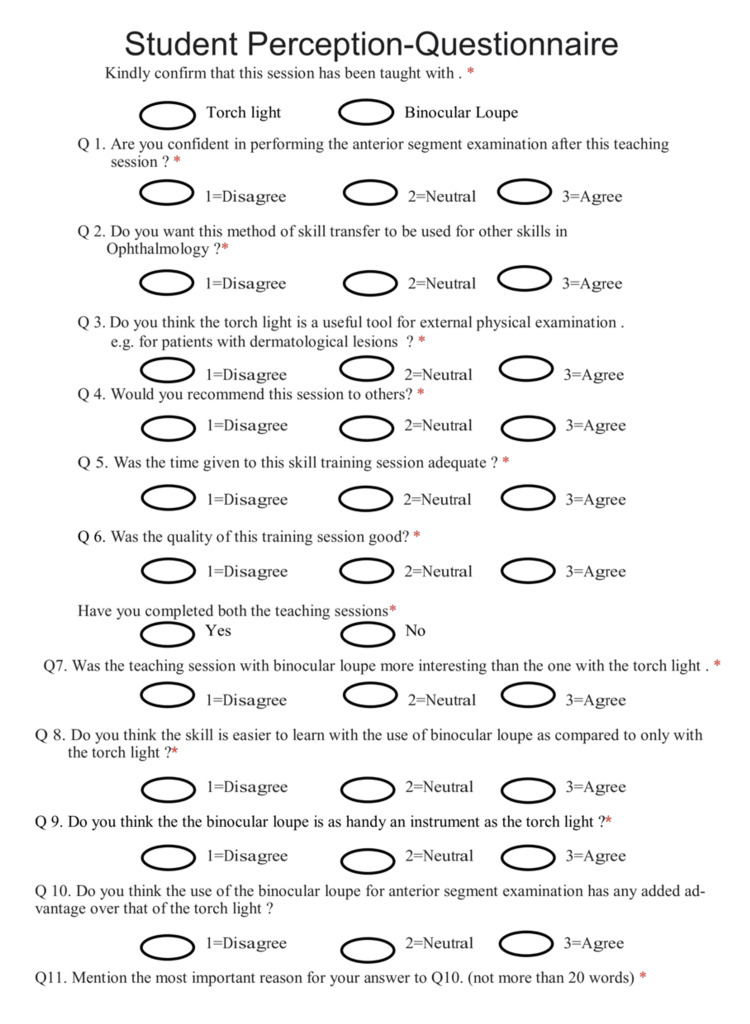
Students' perception questionnaire.

**Figure 6:**
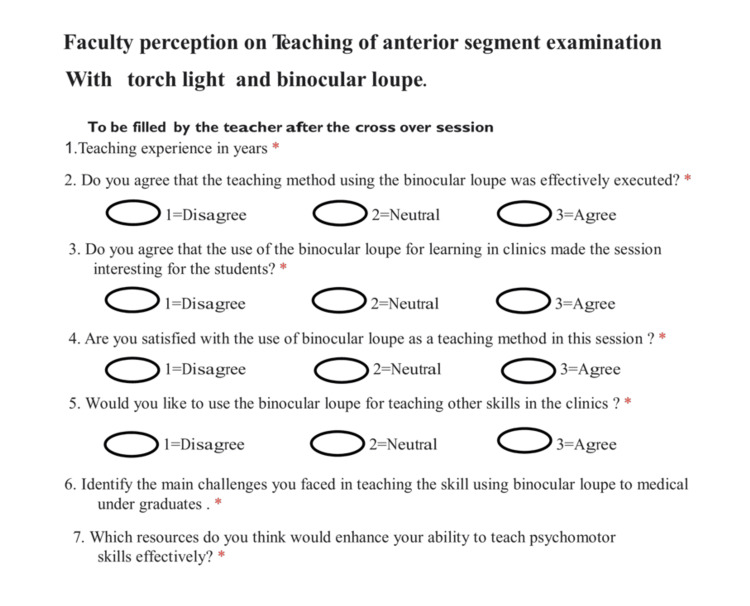
Faculty perception questionnaire.

## Appendices

Appendix A 

Appendix B 
